# Body balance at static posturography in vestibular migraine^☆^

**DOI:** 10.1016/j.bjorl.2017.12.001

**Published:** 2017-12-27

**Authors:** Leslie Palma Gorski, Adriana Marques da Silva, Flávia Salvaterra Cusin, Suelen Cesaroni, Mauricio Malavasi Ganança, Heloisa Helena Caovilla

**Affiliations:** Universidade Federal de São Paulo (UNIFESP), Escola Paulista de Medicina (EPM), São Paulo, SP, Brazil

**Keywords:** Migraine disorders, Dizziness, Postural balance, Vestibular function tests, Transtornos de enxaqueca, Tontura, Equilíbrio postural, Testes de função vestibular

## Abstract

**Introduction:**

Migraine is one of the most frequent and incapacitating headaches, with a high degree of impairment in quality of life. Its association with vestibular symptoms is common, including imbalance and postural instability.

**Objective:**

To evaluate the body balance of patients with vestibular migraine through a static posturography test.

**Methods:**

An experimental group of 31 patients with a medical diagnosis of vestibular migraine in the intercritical period of the disease, and a control group of 31 healthy individuals, matched for age and gender, were submitted to the eight sensory conditions of the Tetrax Interactive Balance System. The parameters analyzed were: stability index, which measures the amount of sway, global stability and ability to compensate postural modifications; weight distribution index, which compares deviations in weight distribution; synchronization index, which measures the symmetry in the weight distribution; postural sway frequency, which indicates the frequency range with more sway; and fall risk index, which expresses the probability of falls.

**Results:**

The stability index was higher in the experimental group in all eight sensory conditions, with a significant difference between the groups in six of them. The weight distribution index was higher in the experimental group in all conditions, with a significant difference in three of them. The number of cases with preferential sway in F2–F4 was significantly higher in the experimental group in three conditions, and in F5–F6 in two, while the fall risk was significantly higher in the experimental group than in the control group.

**Conclusion:**

Patients with vestibular migraine showed compromised body balance at the static posturography test.

## Resumo

**Introdução:**

Migrânea é uma das cefaleias mais frequentes, incapacitante e com elevado grau de comprometimento na qualidade de vida. É comum sua associação a sintomas vestibulares, inclusive desequilíbrio e instabilidade postural.

**Objetivo:**

Avaliar o equilíbrio corporal de pacientes com migrânea vestibular por meio de uma posturografia estática.

**Método:**

Foram submetidos às oito condições sensoriais da posturografia do *Tetrax Interactive Balance System* um grupo experimental de 31 pacientes com diagnóstico médico de migrânea vestibular no período intercrítico da afecção e um grupo controle de 31 indivíduos hígidos pareado quanto à idade e sexo. Os parâmetros analisados foram: índice de estabilidade, que mede a quantidade de oscilação, estabilidade global e habilidade para compensar modificações posturais; índice de distribuição de peso, que compara os desvios na distribuição do peso; índice de sincronização, que mede a simetria na distribuição de peso; frequência de oscilação postural, que aponta a faixa de frequência com mais oscilação; e índice de risco de queda, que expressa à probabilidade de ocorrerem quedas.

**Resultados:**

O índice de estabilidade foi maior no grupo experimental em todas as oito condições sensoriais, com diferença significante entre os grupos em seis delas; o índice de distribuição de peso foi maior no grupo experimental em todas as condições, com diferença significante em três delas; o número de casos com oscilação preferencial em F2-F4 foi significantemente maior no grupo experimental em três condições, e, em F5-F6, em duas; o índice de risco de queda foi significantemente maior no grupo experimental do que no grupo controle.

**Conclusão:**

Pacientes com migrânea vestibular apresentam comprometimento do equilíbrio corporal à posturografia estática.

## Introduction

Migraine is a Greek term that means “half of the skull”. It is also known as migraine headache, which is one of the most frequent and incapacitating types of headaches, with a high degree of impairment in quality of life.^1^ It is a primary head pain disorder, as it has no demonstrable cause in usual clinical or laboratory tests, occurs in recurrent episodes lasting from minutes to hours, is unilateral, pulsatile, of moderate or severe intensity, aggravated by physical activities and associated with nausea and/or photophobia and phonophobia.^2^

Patients with vestibulopathies often report migraine, in the same way that patients with migraine usually report dizziness and vertigo; epidemiological studies indicate a causal association between vertigo/dizziness and migraine.^3^

The prevalence of vestibular migraine is variable, according to the diagnostic criteria and the selected population.^4^, ^5^ It was estimated that the prevalence of vestibular migraine in the general population throughout life is approximately 1%.^6^ Using the recent criteria, vestibular migraine was identified in 4.2% of cases, and vestibular migraine was probable in 5.7% in a tertiary referral otorhinolaryngology clinic,^4^ while a multicenter study in neurological clinics identified the prevalence of vestibular migraine as being 10.3% of migraine patients and probable migraine in 2.5%.^7^

The first recurrent migraine-related vertigo attack can occur at any age, but it is most common in women between 30 and 50 years of age – the gender most affected by the condition – whereas it usually has a peak around the age of 40 years in men.^8^

The criteria initially formulated for the clinical diagnosis of migraine related to vestibular dysfunction^3^ were later revised,^2^, ^9^ considering two clinical conditions: vestibular migraine and probable vestibular migraine. The diagnostic hypothesis was established by recurrent vestibular symptoms, history of migraine, a temporal association between migraine symptoms and vertiginous episodes, and the exclusion of other causes. Vestibular symptoms may be spontaneous, positional vertigo due to intolerance to head movements and/or induced by visual stimuli or dizziness, defined as spatial orientation disorder, induced by head movements, with or without nausea. Acute episodes can be of moderate to severe intensity, lasting from five minutes to three days photophobia and auras should be investigated, due to the frequent absence of headache during the condition attacks.^9^, ^10^

When comparing the new criteria with the old ones, the number of patients diagnosed with vestibular migraine decreased, especially considering the characterization of type, intensity and duration of dizziness, making the diagnosis more specific, although less sensitive.^11^

In our country, an epidemiological study of 85 patients diagnosed with vestibular migraine showed that 94.1% were females, with a mean age of 46.1 years. Vertigo and headache were concomitant in most cases, and the headache appeared on average 7.3 years before the vestibular symptoms.^12^ Other symptoms reported by patients with migraine and vestibular dysfunction are imbalance and postural instability.^8^, ^13^ In 147 patients with vestibular migraine, instability was reported by 91% of cases and imbalance by 82%.^14^

Postural imbalance and instability can be investigated through static and dynamic posturography. The Tetrax Interactive Balance System™ (Tetrax IBS™) is a static posturography test consisting of four integrated plates that measure postural sway in several sensory conditions. The equipment measures and compares the values of the anterior and posterior parts of each foot (toes and heels) and of each heel with the anterior part of the contralateral foot, through the pressure difference exerted on each plate,^15^ indicates the influence of different systems that affect body balance maintenance, calculates the risk that the individual has to fall and allows monitoring the patient during the course of the treatment.^16^

Posturography studies with the old diagnostic criteria showed that patients with migraine and vestibular dysfunction may show signs of alterations when using vestibular, somatosensory and visual cues to maintain body balance.^13^, ^17^, ^18^, ^19^, ^20^ The present study is justified in patients with vestibular migraine, since this is a complex and frequent condition, with recently established diagnostic criteria, small number of studies assessing body balance and lack of research using the Tetrax IBS™ posturography test according to the current concept.

The objective of this study is to evaluate the body balance of patients with vestibular migraine through static posturography.

## Methods

This descriptive and analytical cross-sectional study was started after its evaluation and approval by the institutional Research Ethics Committee under number 542.408. All individuals were informed about the performed procedures and signed the Free and Informed Consent Form, allowing their participation in the study and subsequent analysis and disclosure of results. Patients who participated in this study were evaluated between 2014 and 2015 in the institution's outpatient clinic.

The experimental group consisted of 31 patients of both genders with a medical diagnosis of vestibular migraine,^9^ in the intercritical period of the disease. These patients were selected sequentially at the first medical consultation, without the influence of interventions such as diet and/or medication use.

The criteria for the clinical diagnosis of vestibular migraine were: (A) at least five episodes of vestibular symptoms, such as spontaneous vertigo or at head movement, positional or induced by visual stimuli, dizziness with head movements with nausea, of moderate to severe intensity, lasting from 5 min to 72 h; (B) current or previous history of migraine with or without aura, according to the International Headache Society criteria; (C) one or more of the following symptoms of migraine in at least 50% of vestibular attacks: migraine-type headache (with at least two of the following characteristics: unilateral, pulsatile, moderate to severe intensity, aggravated by physical activity); photophobia and phonophobia; visual aura; (D) exclusion of other causes.^9^

The control group, matched for age and gender in relation to the experimental group, consisted of 31 healthy individuals from the community, such as patients’ companions, post-graduate students and teachers of the institution. The inclusion criteria for this group were: being healthy; absence of a history of vestibular, auditory, body imbalance and/or headache symptoms; and absence of symptoms or signs of neurological diseases and other conditions. Individuals unable to understand and respond to simple verbal commands, those with psychiatric disorders, those unable to independently maintain the orthostatic position, with severe visual impairment or non-compensated with corrective lenses, orthopedic disorders that cause movement impairment, use of prostheses in the lower limbs and those submitted to body balance rehabilitation in the last six months prior to the study were excluded from the research.

**Figure 1:**
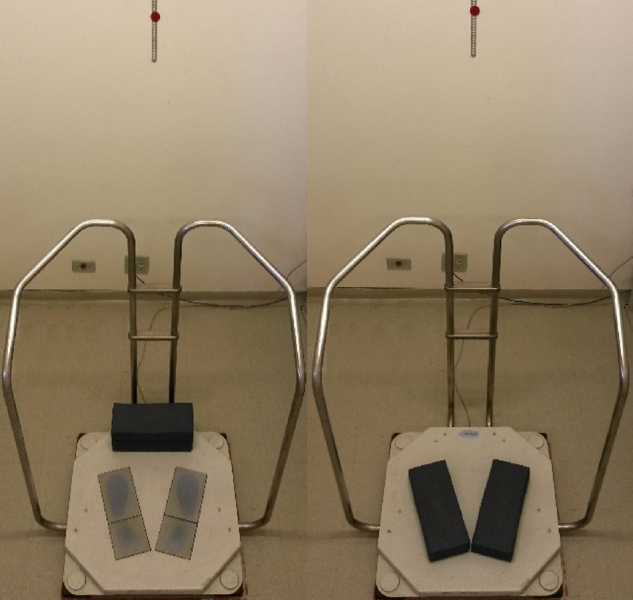
Tetrax Interactive Balance System (Tetrax IBS™) equipment.

The participants were submitted to an evaluation consisting of anamnesis and static posturography test. The anamnesis was performed through a detailed interview, by applying a guided questionnaire on the patients’ clinical history, and the static posturography test was performed using the Tetrax IBS™ system from Sunlight Medical Ltd., Tel Aviv, Israel. The Tetrax IBS™ consists of a specific program installed in a computer, a platform with four integrated, but independent plates (A-B-C-D), which capture variations in weight distribution, handrails and foam mattresses. The platform was supported on firm, leveled floor, without carpet; a target was positioned at eye level placed one meter ahead of the individual being evaluated (Fig. 1).

The subjects were barefoot, resting their toes and heels on the sign indicated on the platform, staring at the target. They were instructed to keep their posture erect and stable, with arms extended along the body for 32 s in each of the following eight sensory conditions: stable surface, eyes open and face forward; stable surface, eyes closed and face forward; unstable surface, eyes open and face forward; unstable surface, eyes closed and face forward; stable surface, eyes closed and head with a 45° rotation to the right; stable surface, eyes closed and head with a 45° rotation to the left; stable surface, eyes closed and head tilted back 30°; and stable surface, eyes closed and head tilted 30° forward.

The sensory condition stable surface, face forward and eyes open is the neutral position, and it analyzes the visual, somatosensory and vestibular systems; the condition stable surface, face forward and eyes closed limits the effect of vision, testing the somatosensory and vestibular systems; the condition unstable surface, face forward and eyes open limits the effect of proprioception, stimulating the visual and vestibular system; the conditions stable surface, head with a 45° rotation to the right and a 45° rotation to the left, eyes closed, eliminate vision and stimulate the vestibular system; and the conditions stable surface, head tilted 30° backwards and head tilted 30° forward, eyes closed, eliminate vision and stimulate the vestibular and cervical systems.^16^

Posturography measured the variations of the vertical force exerted by the heels and tip of the toes, allowing the characterization of body sway according to the displacement of the individual's pressure center. Tetrax IBS™ evaluated the indexes of stability, weight distribution, synchronization of right/left and toe/heel postural sway, F1 to F8 frequencies and F1, F2–F4, F5–F6, F7–F8 frequency ranges of the postural sway in each of the eight sensory conditions, and the risk of fall index.^16^, ^21^

The stability index mathematically indicated the overall stability and ability to compensate postural changes, and assessed the amount of sways on the four platforms according to body weight using the following equation: ST = *t*{∑*n*1[(*an* − *na* − 1)2 + (*bn* − *bn* − 1)2 + (*cn* − *cn* − 1)2 + (*dn* − *dn* − 1)2]}1/2/*W*.*N*; with *a*, *b*, *c* and *d* being the four pressure transducers, *W* the body weight, *t* the experimental time and *n*, the number of signals sampled at 34 Hz. Because it is the average of the sway recorded by each plate, the higher the score, the lower the stability.^16^

The weight distribution index was calculated based on the weight recorded on each of the four plates. The weight distribution deviations in each platform were compared with an expected mean value of 25%; the higher the value, the greater the abnormal weight distribution on each platform.^16^

The synchronization rates of the right-left and heel/toe postural sway measured the coordination between the lower limbs and the weight distribution symmetry. For each condition, six synchronizations were measured: between the heels and toes of each foot (AB, CD); between the two heels and the toes of both feet (AC, BD) and the two diagonals; between the heel of one foot with the contralateral foot toes (AD, BC). The synchronization indexes AB, CD, AD and CB are negative, and the BD and AC, positive. Values with inverted signs suggest excessive postural sway; low values indicate impairment; high values may be due to postural rigidity or intentional lateral sway simulation.^16^

The frequencies of postural sway vary within a spectrum between 0.01 and 3.0 Hz. They were measured through the Fourier Transform, a mathematical treatment of wave signals that indicates the intensity of postural sway at different frequencies and determines the body sway frequency in relation to the most common horizontal plane to maintain the vertical position. The four signals measured at the four sensors were analyzed, and the Tetrax IBS™ recalculated the mean of the results, providing a single value and determining which frequency was the most repeated one. Tetrax IBS™ subdivided the spectrum of postural sway into four frequency bands: low (F1), below 0.1 Hz; medium-low (F2–F4), between 0.1 and 0.5 Hz; medium-high (F5–F6), between 0.5 and 1.0 Hz; and high (F7–F8), above 1.0 Hz.

Excessive postural sways are suggestive of disorders or compensatory attempts. Each frequency range of postural sway emphasizes the use of a certain postural subsystem. The prevalence of postural sways suggests: in the low frequency range, postural control and integrity of vestibular-visual-otolytic systems; in the medium-low frequency range, peripheral vestibular dysfunction, fatigue or physical exhaustion, alcohol intoxication; in the medium-high frequency range, somatosensory reactions mediated by the motor system of the lower limbs and spine; and, in the high frequency range, central nervous system impairment.^16^

The fall risk index, expressed as percentage and variable between zero and 100, analyzed the results of the Tetrax IBS™ parameters in the eight sensory conditions. A value between zero and 36% is considered low risk (marked in green on the Tetrax IBS chart); a value between 37% and 58%, moderate risk (in yellow); and between 59% and 100%, high risk (in red). The higher the score, the greater the risk of falls.^16^

The absence of a probable number or percentage of cases of the population with vestibular migraine and the lack of posturography studies with the Tetrax IBS™ system in individuals with the disease, according to the new diagnostic criteria^9^ in the beginning of the present study, made it impossible to perform a sample calculation. For this reason, a convenience sample was utilized, by sequentially selecting all new cases of vestibular migraine treated at the outpatient clinic in a two-year period.

All data were submitted to descriptive statistical analysis to characterize the sample. Student's *t*-test was used to compare the means of the control and experimental groups regarding age, and the Chi-square test was used to analyze gender homogeneity between the control and experimental groups. The Shapiro–Wilks test was applied to verify data normality. In the comparative analysis of the experimental and control groups, the non-parametric Mann–Whitney test was used for the stability index, synchronization indexes of right/left and toe/heel postural sway, frequency ranges of postural sway and the fall risk index average; Student's *t*-test, for independent samples, was used for the weight distribution index; the extension of Fisher's Exact test and the Chi-square test were used for the distribution of cases according to the standard postural sway score and the frequency range; and the extension of the Fisher's Exact test was used to compare the number of cases according to the degree of fall risk index.

Data were presented as frequencies (relative and absolute), mean, standard deviation, median and minimum and maximum values. The level of significance was set at 5% (*α* = 0.05). The Statistical Package for Social Sciences program (SPSS, version 19) was used for calculations.

## Results

During the data collection period, 31 patients with a diagnosis of vestibular migraine were referred by the otorhinolaryngologist. None of these patients were excluded, as they met the selection criteria. Two individuals were excluded from the control group, as they had vestibular symptoms. The final sample consisted of 31 patients in the experimental group and 31 in the control group.

Of the 31 patients in the experimental group with vestibular migraine, 29 (93.55%) were females and 2 (6.45%) were males; the 31 cases of the control group had the same gender distribution. The group with vestibular migraine ranged in age between 14 and 74 years (mean age of 41.52 years, standard deviation of 14.70 years), and the control group, between 13 and 78 years (mean of 41.03 years; standard deviation of 15.34 years). The groups were considered homogeneous regarding gender (*p* > 0.999) and age (*p* = 0.900).

Table 1 shows the descriptive values and the comparative analysis of the stability index and weight distribution index in the experimental group and the control group in the Tetrax IBS™. The stability index was higher in the group with vestibular migraine than in the control group in all sensory conditions, with a statistically significant difference in six of the eight conditions: eyes open and stable surface; eyes closed and stable surface; eyes open and unstable surface; eyes closed, head to the right and stable surface; eyes closed, head to the left and stable surface; and eyes closed, head tilted back and stable surface. The weight distribution index was higher in the group with vestibular migraine than in the control group in all sensory conditions, with a statistically significant difference in the conditions of eyes open and stable surface; eyes closed, head tilted back and stable surface; and eyes closed, head forward and stable surface.

**Table 1:** Descriptive values and comparative analysis of the stability index and the weight distribution index in the sensory conditions of the Tetrax Interactive Balance System (Tetrax™) in 31 individuals from the control group and 31 from the vestibular migraine group.

Sensory conditions	Stability index			Weight distribution index		
	VM	Control	*p*-Value	VM	Control	*p*-Value
NO	14.04 ± 4.36	11.86 ± 2.39	0.026^a^^,^^c^	6.46 ± 2.70	5.01 ± 2.18	0.023^b^^,^^c^
NC	21.47 ± 9.17	16.01 ± 4.41	0.013^a^^,^^c^	5.72 ± 2.70	4.91 ± 1.87	0.175
PO	24.44 ± 13.60	17.01 ± 4.24	0.015^a^^,^^c^	5.22 ± 2.69	5.13 ± 2.52	0.890
PC	30.59 ± 11.04	26.98 ± 5.72	0.251	4.48 ± 2.21	4.43 ± 2.17	0.937
HR	20.66 ± 10.38	15.80 ± 4.67	0.023^a^^,^^c^	6.24 ± 2.85	5.14 ± 2.23	0.096
HL	20.06 ± 7.62	15.41 ± 5.00	0.011^a^^,^^c^	6.81 ± 2.99	5.61 ± 1.99	0.067
HB	20.81 ± 8.79	16.07 ± 4.74	0.014^a^^,^^c^	6.35 ± 2.86	5.02 ± 2.28	0.047^b^^,^^c^
HF	19.60 ± 8.61	16.44 ± 5.36	0.157	7.38 ± 3.01	5.66 ± 2.33	0.014^b^^,^^c^

Values presented as mean ± standard deviation.

VM, vestibular migraine; NO, stable surface, eyes open and face forward; NC, stable surface, eyes closed and face forward; PO, unstable surface, eyes open and face forward; PC, unstable surface, eyes closed and face forward; HR, stable surface, eyes closed and head with a 45° rotation to the right; HL, stable surface, eyes closed and head with a 45° rotation to the left; HB, stable surface, eyes closed and head tilted back 30°; and HF, stable surface, eyes closed and head tilted 30° forward.

a Mann–Whitney test.

b Student's *t*-test.

c Statistically significant difference between groups (*p* < 0.05).

There was no statistically significant difference between the experimental groups and the control group in the Tetrax IBS™ regarding the synchronization index of right/left and toe/heel postural sways in the eight evaluated sensory conditions, as shown in Table 2.

**Table 2:** Descriptive values and comparative analysis of the synchronization indexes in the eight sensory conditions of the Tetrax Interactive Balance System (Tetrax™) in 31 individuals in the control group and 31 in the vestibular migraine group.

Sensory conditions	AB		CD		AC	
	VM	Control	VM	Control	VM	Control
NO	−733.80 ± 227.64	−741.81 ± 226.54	−750.82 ± 189.17	−761.89 ± 189.25	464.04 ± 330.49	517.34 ± 314.29
NC	−861.50 ± 96.19	−785.48 ± 264.07	−840.24 ± 172.65	−852.80 ± 105.22	697.23 ± 203.59	588.54 ± 322.75
PO	−809.84 ± 160.91	−748.10 ± 181.44	−800.07 ± 174.96	−754.17 ± 237.31	669.88 ± 274.42	666.45 ± 262.35
PC	−808.02 ± 165.78	−801.62 ± 120.94	−807.13 ± 188.99	−817.17 ± 157.11	698.55 ± 231.07	725.57 ± 212.77
HR	−861.25 ± 121.39	−782.49 ± 266.40	−829.65 ± 183.70	−818.23 ± 168.36	621.89 ± 265.34	557.52 ± 299.27
HL	−837.77 ± 128.12	−821.64 ± 146.73	−826.75 ± 168.66	−846.26 ± 127.02	589.09 ± 272.34	639.70 ± 233.80
HB	−856.52 ± 124.64	−832.30 ± 159.06	−835.10 ± 141.58	−870.01 ± 113.17	616.61 ± 278.61	637.17 ± 282.05
HF	−835.29 ± 151.94	−844.11 ± 139.36	−798.33 ± 152.97	−864.97 ± 99.55	530.37 ± 277.84	630.41 ± 228.02

Sensory conditions	BD		AD		BC	
	VM	Control	VM	Control	VM	Control
NO	744.44 ± 249.16	753.56 ± 176.51	−840.47 ± 110.97	−866.00 ± 125.64	−810.87 ± 209.71	−839.05 ± 139.88
NC	858.08 ± 89.52	850.80 ± 92.79	−917.50 ± 64.36	−850.55 ± 169.44	−901.78 ± 113.28	−881.21 ± 130.55
PO	761.89 ± 193.52	677.89 ± 262.53	−899.16 ± 104.39	−890.97 ± 132.12	−898.44 ± 114.41	−906.54 ± 85.86
PC	784.90 ± 171.84	764.08 ± 203.54	−921.56 ± 54.71	−928.62 ± 64.08	−927.15 ± 43.34	−923.27 ± 69.08
HR	858.87 ± 122.66	801.88 ± 218.61	−885.73 ± 66.28	−846.86 ± 143.05	−860.56 ± 121.36	−867.09 ± 120.32
HL	839.10 ± 113.69	802.65 ± 140.63	−853.90 ± 163.42	−866.81 ± 123.33	−863.04 ± 101.03	−875.79 ± 117.39
HB	853.20 ± 105.60	843.56 ± 132.22	−884.64 ± 101.33	−858.92 ± 151.72	−863.95 ± 118.16	−878.72 ± 125.04
HF	827.92 ± 101.26	827.83 ± 149.46	−847.23 ± 133.92	−859.30 ± 120.05	−819.45 ± 146.98	−861.17 ± 160.82

Values shown as mean ± standard deviation.

VM, vestibular migraine; AB, synchronization index between the platforms referring to the toes and heel of the left foot; CD, synchronization index between right toe and heel; AC, synchronization index between the two heels; BD, synchronization index between the two anterior foot parts; AD, synchronization index between left heel and right toes; BC, synchronization index between left toes and right heel; NO, stable surface, eyes open and face forward; NC, stable surface, eyes closed and face forward; PO, unstable surface, eyes open and face forward; PC, unstable surface, eyes closed and face forward; HR, stable surface, eyes closed and head with a 45° rotation to the right; HL, stable surface, eyes closed and head with a 45° rotation to the left; HB, stable surface, eyes closed and head tilted back 30°; and HF, stable surface, eyes closed and head tilted 30° forward.

Mann–Whitney test.

Table 3 shows the distribution of patients according to the standard postural sway score and the frequency range in the eight sensorial conditions in the Tetrax IBS™. The number of cases with worse performance was significantly higher in the group of patients with vestibular migraine in the medium-low frequencies (F2–F4), in the following conditions: eyes closed, head forward and stable surface, eyes closed, head tilted back and stable surface and eyes closed, head to the left and stable surface; and in the medium-high frequencies (F5–F6), in the following conditions: eyes open and unstable surface and eyes closed and stable surface, in comparison with the control group.

**Table 3:** Distribution of patients according to the postural sway standard score and the frequency range and comparative analysis between the control group and the vestibular migraine group in the eight sensory conditions of the Tetrax Interactive Balance System (TetraxTM).

Sensory conditions	Postural sway standard score	Frequency ranges											
		F1			F2-F4			F5-F6			F7-F8		
		VM	C	*p*	VM	C	*p*	VM	C	*p*	VM	C	*p*
**NO**	< 1.5	29	31	0.355^a^	27	30	0.287^a^	24	29	0.126^a^	28	30	0.496^a^
	1.5 – 3.0	1	0		2	1		4	2		2	1	
	3.0 – 6.0	1	0		2	0		3	0		1	0	
	> 6	0	0		0	0		0	0		0	0	
**NC**	< 1.5	30	30	0.601^a^	21	27	0.206^a^	22	31	0.014^a^^,^*	21	26	0.128^a^
	1.5 – 3.0	1	1		7	4		5	0		4	4	
	3.0 – 6.0	0	0		1	0		1	0		6	1	
	> 6	0	0		2	0		3	0		0	0	
**PO**	< 1.5	29	31	0.355^a^	22	27	0.141^a^	25	31	0.023^b^^,^*	30	31	1.000^b^
	1.5 – 3.0	1	0		6	4		6	0		1	0	
	3.0 – 6.0	1	0		3	0		0	0		0	0	
	> 6	0	0		0	0		0	0		0	0	
**PC**	< 1.5	29	27	0.671^**^	23	26	0.320^a^	22	29	0.062^a^	26	27	0.600^a^
	1.5 – 3.0	2	4		6	5		8	2		4	2	
	3.0 – 6.0	0	0		2	0		1	0		1	2	
	> 6	0	0		0	0		0	0		0	0	
**HR**	< 1.5	29	31	0.491^b^^,**^	20	24	0.367^a^	27	31	0.117^a^	24	26	0.582^a^
	1.5 – 3.0	2	0		6	6		3	0		4	4	
	3.0 – 6.0	0	0		4	1		0	0		3	1	
	> 6	0	0		1	0		1	0		0	0	
**HL**	< 1.5	30	30	0.367^a^	18	27	0.029^a^^,^*	26	30	0.213^a^	29	29	0.513^a^
	1.5 – 3.0	1	0		11	4		4	1		2	1	
	3.0 – 6.0	0	1		2	0		0	0		0	1	
	> 6	0	0		0	0		1	0		0	0	
**HB**	< 1.5	31	30	0.671^b^	21	29	0.029^a^^,^*	21	28	0.071^a^	27	27	0.765^a^
	1.5 – 3.0	0	1		7	2		8	3		2	3	
	3.0 – 6.0	0	0		3	0		2	0		2	1	
	> 6	0	0		0	0		0	0		0	0	
**HF**	< 1.5	29	27	0.671^b^	17	28	0.017^a^^,^*	28	29	0.502^a^	28	28	0.716^a^
	1.5 – 3.0	2	4		12	3		1	2		2	1	
	3.0 – 6.0	0	0		1	0		1	0		1	2	
	> 6	0	0		1	0		1	0		0	0	

VM, Vestibular Migraine; C, Control; NO, stable surface, eyes open and face forward; NC, stable surface, eyes closed and face forward; PO, unstable surface, eyes open and face forward; PC, unstable surface, eyes closed and face forward; HR, stable surface, eyes closed and head with a 45° rotation to the right; HL, stable surface, eyes closed and head with a 45° rotation to the left; HB, stable surface, eyes closed and head tilted 30° backwards; HF, stable surface, eyes closed and head tilted 30° forward.

a Chi-square test.

b Extension of Fisher's exact test.

* Statistically significant difference between groups (*p* ≤ 0.05).

Table 4 shows the descriptive values and the comparative analysis of the fall risk index in the control and the experimental groups in the Tetrax IBS™. The group with vestibular migraine had a higher fall risk than the control group, with a statistically significant difference. The fall risk was on average of a mild degree in both groups.

**Table 4:** Descriptive values and comparative analysis of the fall risk in the sensory conditions of the Tetrax Interactive Balance System (Tetrax™) in 31 individuals of the control group and 31 of the group with vestibular migraine.

Groups	Fall risk					
	Mean	Standard deviation	Minimum value	Median	Maximum value	*p*-Value
VM	33.29	23.55	2	30	96	0.002^a^
Control	17.68	11.34	2	14	48	

VM, vestibular migraine.

Mann–Whitney test.

a Statistically significant difference between the groups (*p* ≤ 0.05).

Table 5 shows the number and percentage of patients according to the degree of fall risk. The proportion of patients with moderate and high fall risk was significantly higher in the vestibular migraine group compared to the control group. A participant in the control group, a 57-year-old female, had a moderate degree of fall risk.

**Table 5:** Distribution of patients according to the degree of fall risk at the Tetrax Interactive Balance System (Tetrax™) in 31 individuals from the control group and 31 from the vestibular migraine group.

Groups	Fall Risk			
	Low *n* (%)	Moderate *n* (%)	High *n* (%)	*p*-Value
VM	19 (61.29%)	8 (25.81%)	4 (12.90%)	0.003^a^
Control	30 (96.77%)	1 (3.23%)	0	

VM, vestibular migraine.

Extension of Fisher's exact test.

a Statistically significant difference between the groups (*p* ≤ 0.05).

## Discussion

The clinical history of patients with vestibular migraine is quite diverse. The condition can occur at all ages, being more frequent in women. In most patients, the migraine precedes vestibular symptoms, and, in some cases, these only occur many years after the migraine attacks ended.^3^, ^8^

In this study, patients with vestibular migraine were aged between 14 and 74 years and there was a prevalence of females, corroborating the findings of other authors, using either the old or the most recent criteria.^7^, ^8^, ^12^, ^18^, ^19^

Imbalance and postural instability were not included in the new criteria for vestibular migraine definition, although they have been mentioned as symptoms that may occur in migraine patients,^8^ including in the absence of vertigo^13^ and with relevant prevalence.^14^

Despite the complaints of imbalance and postural instability in patients with vestibular migraine, there have been few clinical investigations with posturography. Some studies, considering the criteria previous to the current ones, observed signs of alterations during the use of vestibular, somatosensory and visual cues to maintain body balance at the posturography test.^13^, ^17^, ^18^, ^19^, ^20^ Postural tests allow identifying subclinical vestibular dysfunctions that can be clinically significant, even in individuals with no history of dizziness and/or vertigo.^22^ Posturography studies in patients with vestibular migraine diagnosed according to the new criterion were not found in the literature,^2^, ^9^ suggesting the original aspect of the present study.

In the Tetrax IBS™ system and other posturography tests, the sensory information is modified or excluded to allow the role identification of each information in body balance maintenance, considering the task of remaining on the force platform in the upright position without moving. The proprioceptive afferents can be modified by placing the patient on an unstable surface (foam) or by changing the neck position (head to the right, to the left, upward and downward); the visual afferents can be excluded (eyes closed), while the vestibular information remains the same in all measurements, except those with the head upward and downward, which modify the position of the otoliths and may produce an additional vestibular stimulation. Disorders in a certain system are detected by comparing the results in the different sensory conditions, with or without the exclusion of sensory information.^16^

The Tetrax IBS™ stability index – which quantifies postural sway and is an indicator of overall stability, expressing the ability to control and compensate postural disturbances – showed increased values in the vestibular migraine group in all eight sensory conditions, significantly in six of them, denoting the patients’ objective postural instability. Two conditions, one with eyes closed and unstable surface and the other with eyes closed, head forward and stable surface, showed similar results between the experimental and control groups, suggesting similar performance regarding the ability to maintain body balance in both groups. In 32% of the non-institutionalized civilian population in the United States aged 40 years or older and with no history of dizziness, there was also difficulty standing with eyes closed on an unstable surface,^22^ corroborating the findings of this research. Tetrax IBS™ in patients with migraine vertigo^20^ also showed increased values in all sensory conditions, although the difference in relation to the control group was significant in only one sensory condition: with eyes open on an unstable surface. It is possible that a higher number of cases in our study and in the studies by other authors would contribute to finding a significant difference between the experimental and the control groups in all sensory conditions evaluated.

The weight distribution index values at the Tetrax IBS™ system were increased in the vestibular migraine group in all evaluated sensory conditions, with a significant difference in three of the eight conditions. Changes in these conditions can be attributed to central nervous system and vestibulocervical disorders, by suppressing the visual system and stimulating the vestibular system and the cervical segment.^16^ However, another study with the Tetrax IBS™ system in 16 patients with migraine vertigo did not identify an increase in the weight distribution index values or a significant difference in comparison with the control group in the evaluated sensory conditions.^20^ The difference between the results of the two studies may be related to the small number of participants in the latter and also to the use of different diagnostic criteria.

The synchronization index values of the right/left and toe/heel postural sway in the control group and the group with vestibular migraine at the Tetrax IBS™ were symmetrical in the eight sensory conditions, indicating the use of adequate compensatory mechanisms and simultaneous activation of the parallel plates of the Tetrax IBS™ platform without excessive postural sway, postural rigidity or intentional simulation^16^ in patients with vestibular migraine. Therefore, we did not find alterations in weight distribution and in the postural sway coordination between the lower limbs at the Tetrax IBS™ system in patients with vestibular migraine. No studies were found in the literature that evaluated the synchronization indexes of postural sway in vestibular migraine.

In this study, when the distribution of patients with vestibular migraine according to the standard postural sway score and frequency ranges was assessed, it was observed that the middle-low frequencies (F2–F4) in three sensory conditions and the middle-high (F5–F6) in two conditions were the ones with the highest number of patients with significantly worse performance, suggesting the prevalence of vestibular and somatosensory dysfunction.^16^ Similar to the findings in this study, another study in patients with migraine vertigo and a control group, identified a significant difference in the middle-low frequencies (F2–F4); however, the study also found a significant difference in the high frequencies (F7–F8), suggesting additional abnormalities in the central nervous system and, consequently, concomitant peripheral and central vestibular involvement.^20^

The mean values of the fall risk in the Tetrax IBS, based on the patients’ performance in the eight assessed conditions, were significantly higher in the group with vestibular migraine, when compared to the control group. When analyzing the fall risk related to the degree, patients with vestibular migraine had a moderate and high-risk prevalence in comparison to the control group, which mostly had a low fall risk; the finding of a moderate fall risk in the elderly from the control group showing altered results in conditions with head position changes^23^ or not^22^ was also observed in healthy individuals older than 40 years. In contrast, another study did not find a significant difference between patients with migraine vertigo and the control group regarding the fall risk, both for mean values and for degree,^20^ which could be explained by the reduced number of patients or the use of different diagnostic criteria.

The high fall risk in individuals with vestibular migraine indicates the need for follow-up of these patients and the use of measures to prevent falls, such as a domestic reorganization, including the removal of objects against which one might stumble, and performing exercises to improve balance.^16^

Despite the assertions that the tests are usually normal,^13^ or non-specific in the intercritical period and do not add much data to the diagnosis,^10^ this study showed significant postural control alterations in patients with vestibular migraine at the posturography. Possible limitations of this study were the selection of patients based on their medical history and the nonperformance of neurological and neurotological evaluation. Moreover, patients were not assessed during acute episodes and no data were collected on their impact or patient disability.

The identification of body balance impairment characteristics in patients with vestibular migraine may have important diagnostic, preventive and therapeutic implications. Changes in posturography parameters should continue to be evaluated in future scientific investigations.

## Conclusion

Patients with vestibular migraine have body balance impairment at the static posturography test.

## Conflicts of interest

The authors declare no conflicts of interest.
